# Rac1 in gastric cancer: a molecular driver of invasion, EMT, and therapeutic resistance

**DOI:** 10.1186/s12967-025-07256-x

**Published:** 2025-11-04

**Authors:** Jianwen Li, Yahong Zhu, Ruifeng Duan, Yueli Tian, Xingang Li, Ying Song

**Affiliations:** 1 Gastroenteric Medicine and Digestive Endoscopy Center, The Second Hospital of Jilin University, Changchun, Jilin 130042 China; 2 School of Medical and Health Sciences, Edith Cowan University, Joondalup, Western Australia 6027 Australia; 3 School of Science, Edith Cowan University, Joondalup, Western Australia 6027 Australia; 4 Nutrition and Health Innovation Research Institute, Edith Cowan University, Joondalup, Western Australia 6027 Australia

**Keywords:** Rac1, Gastric cancer, Rho GTPases, Signaling pathways, Molecular inhibitors, miRNA, Immunotherapy

## Abstract

Gastric cancer (GC) ranks as the fifth most common cancer worldwide and is the third main cause of cancer-related mortality, posing a substantial burden to global public health. Research suggests that targeted therapy and immunotherapy may become more effective treatment options for advanced, unresectable, or metastatic gastric cancer. Ras-related C3 botulinum toxin substrate 1 (Rac1), a small GTP-binding protein within the Rac subfamily of the Rho GTPase family, is a critical molecule that promotes cancer cell invasion and metastasis by regulating signal transmission and promoting cell polarity. It has emerged as a key driver of tumor development and metastasis in several malignancies, including breast, lung, prostate, ovarian, gastric, and pancreatic cancers. This review summarizes the structure, regulatory dynamics, and signaling mechanisms of Rac1 in gastric cancer growth, epithelial-to-mesenchymal transition (EMT), and metastasis, as well as the roles of factors such as hypoxia, oxidative stress, and H. pylori infection. Additionally, it highlights small-molecule inhibitors targeting Rac1, miRNAs capable of suppressing Rac1, and ongoing research on Rac1-related immunotherapy. The potential of Rac1 as a therapeutic biomarker in gastric cancer and the remaining challenges in this area are also discussed. This review advances the understanding of Rac1’s role in gastric cancer, provides a theoretical foundation for further studies, and supports the development of precision medicine for this disease.

## Introduction

Gastric cancer (GC) significantly contributes to global cancer-related mortality, with its subtle progression often leading to late-stage diagnosis, which significantly limits treatment options [1–3]. While molecularly targeted therapies (e.g., HER2/Claudin18.2 inhibitors) combined with platinum regimens have improved survival, tumor microenvironment-driven resistance and biomarker-defined patient stratification continue to limit clinical outcomes [4–7]. The challenges require a thorough investigation of signaling regulators in the pathogenesis of gastric cancer.

**Fig. 1:**
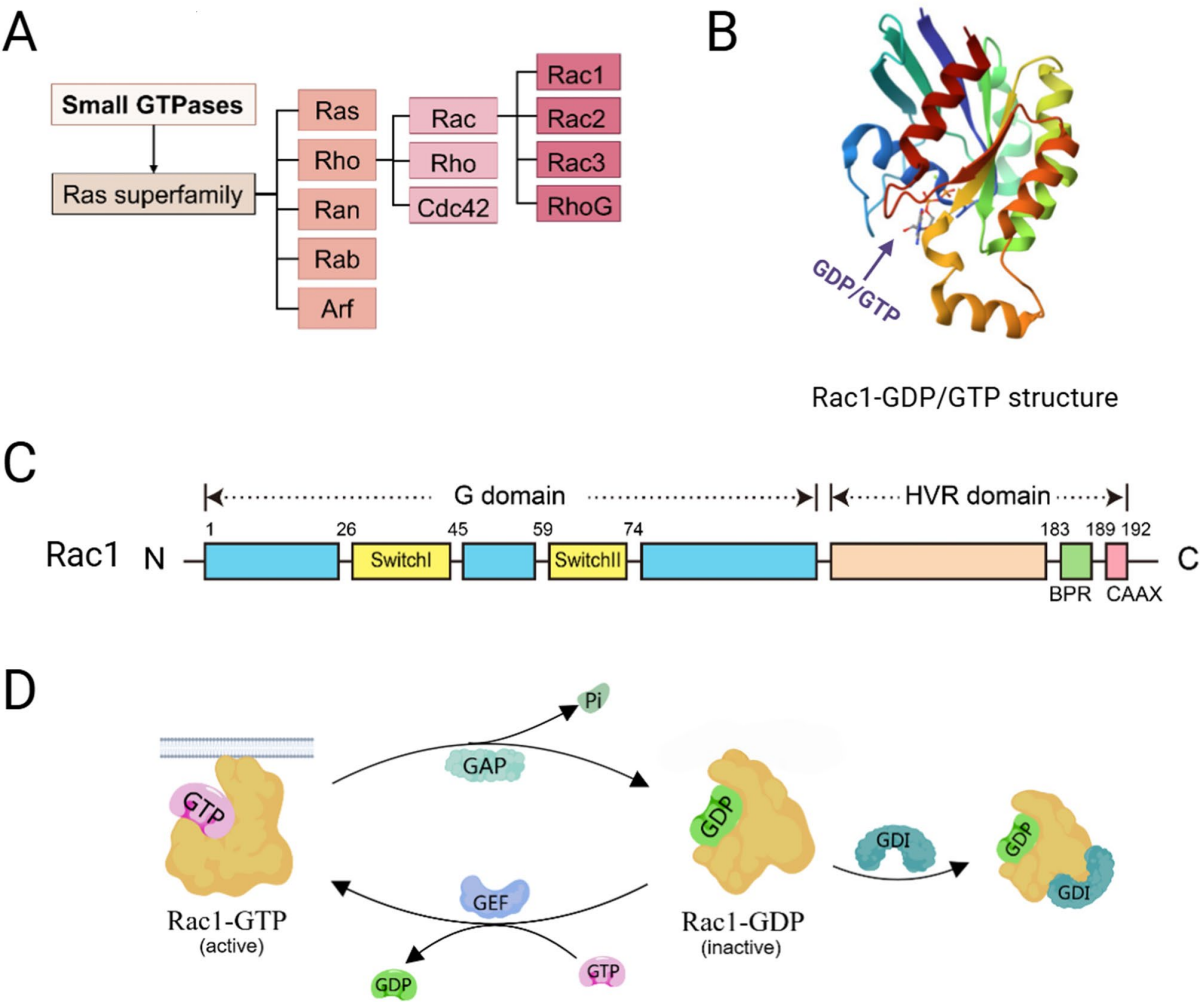
Structural classification and regulatory mechanisms of Rac1 as a small GTPase. **(A)** Classification of small GTPases. **(B)** The molecular model of Rac1 protein with the GTP/GDP binding site. **(C)** The molecular structure of Rac1. **(D)** Conversion between Rac1-GTP and Rac1-GDP and regulation by GEF, GAP, and GDI

Rho guanosine triphosphatases (Rho GTPases), essential regulators of cytoskeletal dynamics, promote cellular processes vital to malignancy (Fig. 1a) [8–10]. Among them, Ras-related C3 botulinum toxin substrate 1 (Rac1) (NP_008839.2) orchestrates actin cytoskeletal dynamics to regulate fundamental cellular processes [11–13]. Extensive studies have confirmed the Rac1 oncogenic function, including gastric cancer [14–16], colon cancer [17, 18], breast cancer [19], lung cancer [20, 21], prostate cancer [22], ovarian cancer [23], and pancreatic cancer [24]. Evidence suggests that hyperactivation and overexpression of Rac1 are associated with gastric cancer in multiple aspects. It drives cancer progression through PI3K/AKT-mediated epithelial-mesenchymal transition (EMT), correlates with deep mural invasion (T3/T4), and metastasis through WAVE/Arp2/3-dependent pseudopod formation [25–27]. Rac1 interacts dynamically with *H. pylori* virulence factors (e.g., LPS, CagA, and VacV) to amplify oxidative stress and genomic instability during gastric carcinogenesis [28].

This review systematically delineates the functional landscape of gastric cancer from molecular mechanisms to therapeutic perspectives. Particular emphasis is placed on Rac1’s interaction network during oncogenesis, invasion, and metastasis, alongside its emerging potential as a predictive biomarker for therapeutic decision-making and prognostic stratification. Exploring deeper into the spatiotemporal regulation of Rac1 signaling cascades in gastric carcinogenesis is expected to enhance prognostic precision and inform the development of biomarker-driven clinical strategies.

## Molecular structure and signaling of Rac1

Rac1 is named for being a substrate of botulinum C3 ADP ribosyltransferase, first discovered in human platelets [29, 30]. Its N-terminus features a conserved G domain (G1-G5), essential for GTP binding. This domain encompasses switches I and II [12]. Switch I engages with GAPs or downstream effectors, whereas switch II interacts with GEFs [31]. The C-terminus contains a hypervariable region with a polybasic region and CAAX box, crucial for post-translational modifications, complex formation, and Rac1’s intracellular localization and kinase interactions (Fig. 1b and c) [12, 32–34].

Like most Rho family proteins, the Rac1 protein function switches between GTP-binding (on) and GDP-binding (off) through the structural changes of switch I and switch II. Three primary types of proteins are associated with Rac1 to facilitate its biological function: guanine nucleotide exchange factors (GEFs), GTPase-activating proteins (GAPs), and guanine nucleotide dissociation inhibitors (GDIs) (Fig. 1d). GEF facilitates the release of GDP, subsequently activating Rac1 to bind to GTP, including members of the Dbl family (Tiam1, Vav, and Trio) and cytokine-specific factors (DOCK). GAP inactivates Rac1 by enhancing its intrinsic GTP-hydrolysis activity [12, 31]. GDI sequesters Rac1 in an inactive state and safeguards it from proteasomal degradation in preparation for subsequent signaling stimuli, which also regulate Rac1’s access to regulatory GEFs and GAPs, effectors, and the membranes where these effectors are located [33, 35].

The activation state and post-translational modifications of Rac1 are closely linked to its varied intracellular localization [33]. Primarily, activated Rac1 is found on the cytoplasmic side of the plasma membrane. When GTP is bound, Rac1 changes conformation, exposing its C-terminus and undergoing lipid modification to form a hydrophobic lipid tail. This tail attaches activated Rac1 to the inner membrane face by inserting into the phospholipid bilayer of the cell membrane. By enlisting and activating downstream effector proteins, it controls cytoskeletal remodeling and other processes [36]. Meanwhile, inactivated Rac1 (Rac1-GDP) is mostly found in the cytoplasm, where it is bound to GDI [33]. Rac1 can also localize to different subcellular compartments, which allows for compartmentalized Rac1 signaling. It contributes to the regulation of endomembrane transport and organelle function when in its activated functional state [37, 38]. 

Rac1 possesses intricate downstream signaling pathways (Table 1). Membrane-bound Rac1-GTP has been shown to recruit p21-activated kinases (PAKs) by binding through the Cdc42-Rac-interacting-binding (CRIB) structural domain. This interaction modulates bistable GTPase activity, cellular morphology, and migratory transitions. PAKs induce cytoskeletal remodeling through the phosphorylation of LIM kinase (LIMK) [39, 40]. LIMK, in turn, phosphorylates actin filament-cutting proteins, releasing them from actin filaments and thereby inhibiting actin-cutting activity. Through this mechanism, the Rac1/Pak1/LIMK1/actin filament-cutting protein axis regulates actin filament turnover at the plate pseudopod [41]. 

 Rac1 can also attach to and activate protein kinase C-related kinase 1 (PRK1), which links with the actin cross-linking protein α-actinin [42]. Additionally, Rac1-GTP directly engaged with signal transducers and activators of transcription 3 (STAT3) to facilitate STAT3 phosphorylation, hence enhancing the EMT of cancer cells [43]. Furthermore, Rac1 activates the Wiskott-Aldrich Syndrome Protein-family verprolin-homologous (WAVE), which exists exclusively in the WAVE Regulatory Complex (WRC), through its interaction with the insulin receptor tyrosine kinase substrate p53 (IRSp53) scaffold molecule. This interaction induces a series of specific conformational changes that facilitate the release of the chelated WH2-central-acidic (WCA) peptide, subsequently promoting actin polymerization via the Arp2/3 complex and initiating the formation of new branching filaments [27, 44].

**Table 1:** Intracellular functions of Rac1

Rac1 binding proteins	Functions	References
PAKs	Induce cytoskeletal rearrangements.	[39, 40]
	Regulate actin filament turnover at pseudopodia.	[41]
PRK1	Interacts with α-actinin to influence actin cross-linking.	[42]
STAT3	Phosphorylation promotes EMT.	[43]
WRC	Initiates the growth of new actin branches via the Arp2/3 complex.	[27, 44]

## Rac1 function and its regulation in gastric cancer

### Rac1 promotes gastric cancer cell proliferation and migration

Rac1 functions as a molecular switch vital for fundamental biological processes, including cell cycle progression, directed migration, and tissue invasion. Overexpression of Rac1 in gastric cancer is mechanistically linked to enhanced tumor proliferation and aggressive metastatic spread.

GEFs, GAPs, and GDIs associated with Rac1 regulation in gastric cancer are shown in Table 2. Research indicates that the atypical Rho GEF DOCK6 facilitates the proliferation, migration, and invasion of gastric cancer cells while also enhancing chemo- and radio-resistance via the DOCK6/Rac1/Cdc42 axis and the DOCK6/WNT/β-catenin signaling pathway [45, 46]. Rho GEF Vav2/Vav3 promotes invasion and metastasis in gastric cancer and is significantly correlated with the expression of multiple matrix metalloproteinases (MMP-2, MMP-9) and tissue inhibitor of metalloproteinases (TIMP-1) [47].

Most GAPs suppress Rac1 activity by promoting its conversion from the active GTP-bound to the inactive GDP-bound state. However, the function of IQ motif-containing GTPase-activating protein 1 (IQGAP1) in gastric cancer is controversial. IQGAP1 utilizes a GRD structural domain that binds to GTP to stabilize the activity of Rac1. A clinical study reported that patients with Tiam1 expression and a lack of IQGAP1 expression had a trend toward favorable prognoses [48]. Meanwhile, animal investigations indicate that the absence of IQGAP1 does not influence tumor formation or progression but may contribute to the conservation of gastric mucosal integrity in older mice [49]. However, another study suggested the opposite, that mice deficient in IQGAP1 showed a higher incidence of gastric cancer after infection with *Helicobacter pylori (H. pylori)* [50]. These differences have been suggested to be possibly related to the study methodology, species, or concentrations [51]. Rho GDP dissociation inhibitor 2 (RhoGDI2) recruits Rac1 to Filamin A (a sizeable cytoskeletal protein), resulting in enhanced interaction between Rac1 and Rho GEF Trio to increase Rac1 activity and promote gastric cancer cell invasion [52]. 

**Table 2:** List of GEFs, GAPs, and GDIs associated with Rac1 regulation in gastric cancer

Classification	Regulatory factor	References
GEF	Vav1/2/3	[47, 53, 54]
	DOCKs	[45, 46, 55–58]
	PREX1/2	[59, 60]
	Tiam1	[61]
	Trio	[62]
	ARHGEF3	[63]
	GEF-H1	[64]
	Son of Sevenless 1 (SOS1)	[65]
GAP	A Rho GAP 15 (ARHGAP15)	[66]
	IQGAP1	[48, 51]
	CdGAP (ARHGAP31)	[67]
	β2-chimaerin	[68]
	ARHGAP24 (FilGAP)	[69, 70]
	CED-12(ELMO)	[71]
	NF1 encodes neurofibromin	[72]
	Regulator of G-protein signaling 4 (RGS4)	[73]
	The human minor histocompatibility antigen 1 (HMHA1)	[74]
	srGAP3	[75]
GDI	RhoGDI2	[52]

Moreover, additional regulatory molecules are also involved. Nectin-4, a cell adhesion molecule highly expressed in gastric cancer, activates Rac1 via the PI3K/AKT signaling pathway, enhancing lamellipodia formation, cellular migration, and proliferation [76, 77]. High expression of certain integrins also facilitates cell adhesion and Rac1 activation. Integrin α5 (ITGA5) activates the FAK/Src/Rac1 pathway, fostering the malignant characteristics of gastric cancer cells and improving adhesion between cells and extracellular matrix proteins [78]. Yu et al. established that integrin αvβ6 (ITGAV: ITGB6) increases the proliferation and invasion of gastric cancer cells by targeting Rac1, as evidenced by bioinformatics analyses and experimental data, and identified it as an independent prognostic risk factor for gastric cancer [14].

Rac1 also influences integrin function. Zhang et al. showed that Rac1 induces abnormal expression and aggregation of the integrin subunit α6 (ITGA6) on the cell membrane via transfection experiments. The interaction between the extracellular matrix (ECM) and ITGA6 subsequently activates the FAK/AKT pathway in gastric cancer cells, exacerbating the peritoneal metastasis of gastric cancer [15]. 

### Rac1 promotes gastric cancer cell EMT

EMT is essential for the invasion and metastasis of epithelial malignancies such as lung, liver, stomach, and colon cancers [79]. In gastric cancer, EMT promotes cancer cell migration in neighboring cells and tissues, transforms polarized epithelial cells into mobile mesenchymal cells, and has a positive effect on tumor invasion and drug resistance [80].

**Fig. 2:**
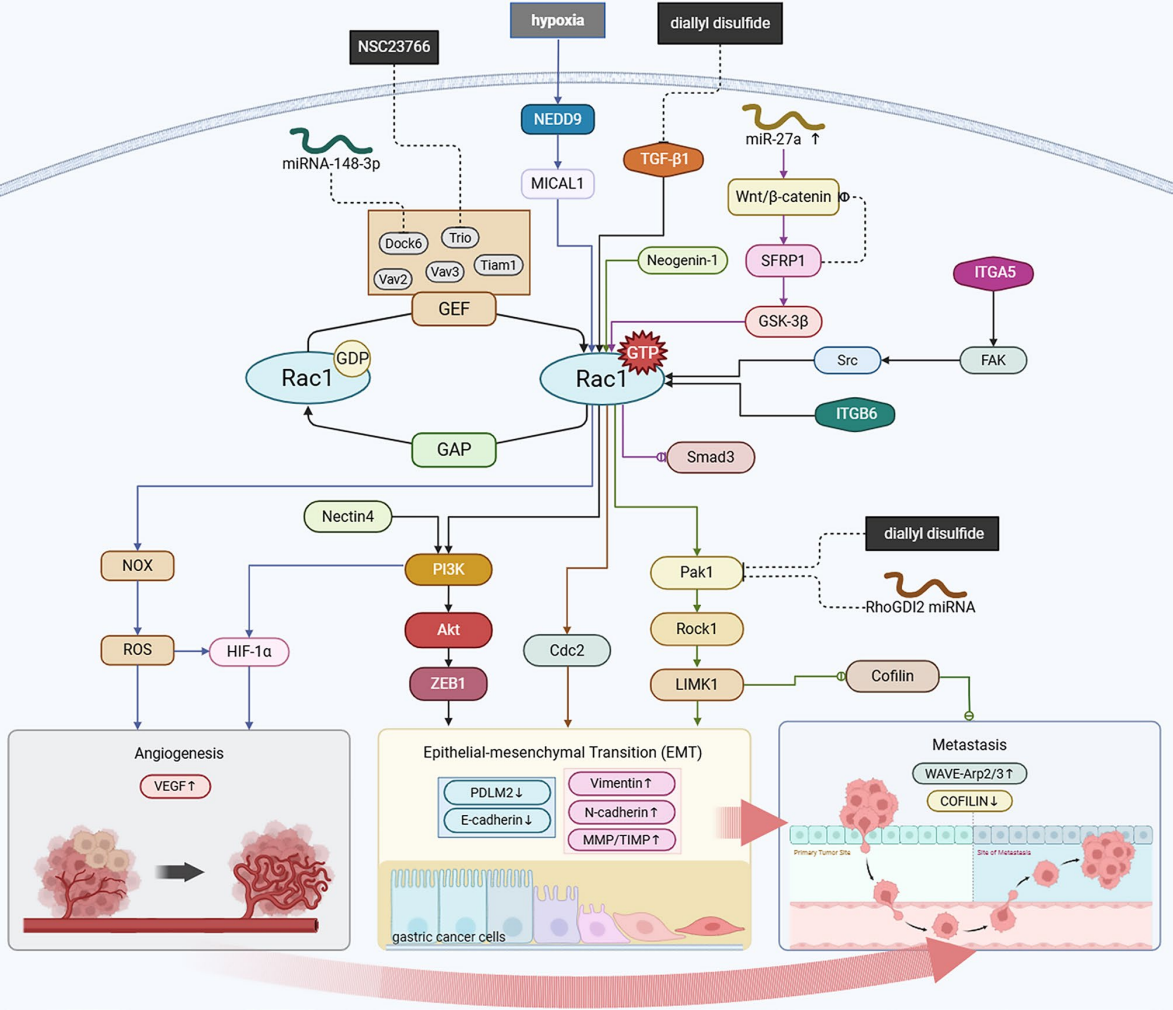
Rac1 pathways in gastric cancer. Schematic illustration of Rac1 signaling pathways and effector functions in gastric cancer

Figure 2 illustrates the role of Rac1 in the regulation of EMT in gastric cancer. Secretory frizzled-related protein 1 (sFRP1) has elevated expression in gastric cancer tissues, with its up-regulation facilitated by the Wnt/β-catenin signaling pathway [81]. sFRP1 reinstates glycogen synthase kinase 3β (GSK3β) activity, thereby facilitating Rac1 activation, while ectopic overexpression of Rac1 concurrently suppresses SMAD family member 3 (Smad3) activity; these interactions collectively enhance the malignant phenotype of EMT in gastric cancer [82]. Su et al. demonstrated that TGF-β1 treatment promotes EMT and invasion in gastric cancer cells, accompanied by elevated expression of Rac1 and β-catenin, effects that can be mitigated by diallyl disulfide [83]. Diallyl disulfide was found to inhibit the Rac1-Pak1/Rock1-LIMK1 pathway, resulting in decreased expression of p-LIMK1 and p-cofilin1, which decreased MMP-9 expression, increased TIMP-3 expression, and inhibited EMT [84].

Gao et al. developed an in vitro model of TGF-β1-induced epithelial-mesenchymal transition in MGC-803 and MKN45 cell lines. Interference with Rac1 and Prex1 was found to inhibit EMT, promote apoptosis, upregulate E-cadherin and PDLIM2 expression, and inhibit N-cadherin and vimentin expression [16]. Furthermore, zinc finger E-box-binding homeobox 1 (ZEB1), ZEB2, and Snail are among the EMT-related proteins that show increased expression in gastric cancer tissues [85, 86]. Several studies show that Rac1 activation increases ZEB1, Snail, and TWIST expression, for example, via the Rac1/PI3K/AKT signaling pathway [25, 87, 88]. These EMT regulators attach to the promoter regions of epithelial genes, such as E-cadherin, enlist co-repressors, and effectively block the transcription of those genes. Concurrently, they stimulate the expression of mesenchymal genes like N-cadherin, which works in concert to support cellular EMT [89].

### Rac1 in gastric cancer cell hypoxia and oxidative stress

Rac1 is implicated in hypoxia and oxidative stress in gastric cancer, facilitating the migration of gastric cancer cells (Fig. 2). Active Rac1 facilitates the production of reactive oxygen species (ROS) through its interaction with the cytoplasmic activator of NADPH oxidase (NOX) [44]. Tatsuya et al. demonstrated that hypoxia markedly elevated the expression of the lipid scavenger cluster of differentiation 36 (CD36) in gastric cancer cells. Furthermore, the increase in active Rac1 and Cdc42 may facilitate the enhanced migratory and invasive capabilities of CD36-overexpressing cells [90]. Asporin (ASPN), a small proteoglycan, mitigates oxidative stress in gastric cancer cells and activates the Rac1 signaling pathway through the upregulation of cluster of differentiation 44 (CD44), thereby enhancing the migratory and invasive capabilities of these cells [91].

Neural precursor cell expressed developmentally downregulated protein 9 (NEDD9) expression increases in gastric cancer cells under hypoxic conditions, which regulates the increased expression of molecule interacting with Cas L1 (MICAL1) and facilitates hypoxia-induced migration of gastric cancer cells in a Rac1-dependent manner [92]. PlexinA1 interacts with the actin cytoskeleton regulator MICAL1 in a manner dependent on Rac1 and ROS, modulates vimentin expression, and prevents MICAL1 degradation, thereby facilitating gastric cancer cell migration [93]. Additionally, a Rho GTPase activating protein 15 (ARHGAP15) inhibits Rac1 activity and reduces intracellular ROS accumulation, thereby improving the antioxidant capacity of tumor cells during oxidative stress [66]. Xue et al. demonstrated that the activation of Rac1 in gastric cancer cells leads to an upregulation of hypoxia-inducible factor (HIF)-1α expression and vascular endothelial growth factor (VEGF), which is associated with the PI3K pathway [94, 95].

### Rac1 in *H. pylori*-associated gastric cancer

**Fig. 3:**
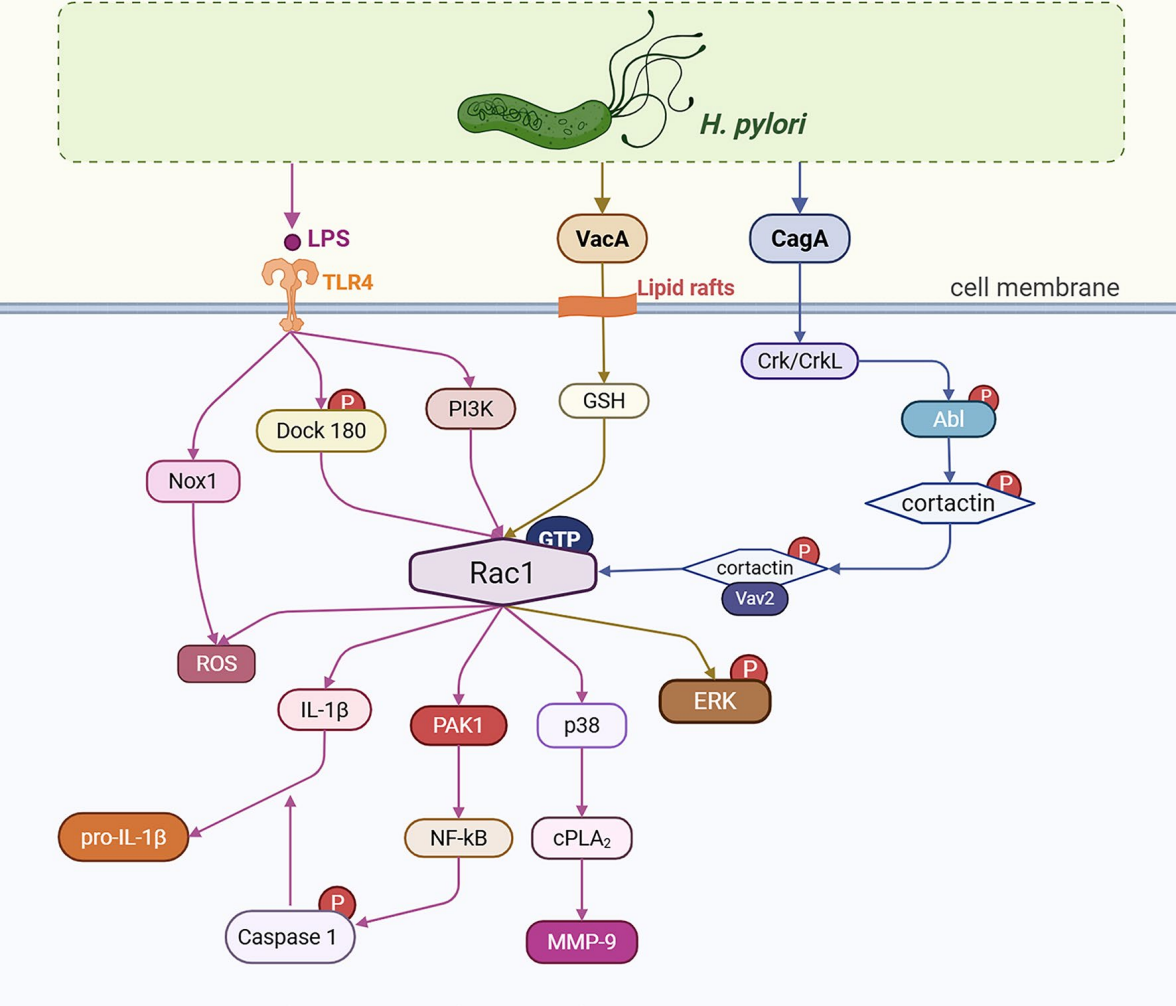
Effect of *Helicobacter pylori* on Rac1 expression. Activation of Rac1 by *Helicobacter pylori*-associated toxins promotes gastric carcinogenesis

*Helicobacter pylori* (*H. pylori*) infection significantly contributes to the Correa sequence and serves as a critical risk factor for gastric invasive carcinoma. Its colonization of the gastric mucosa activates the MAPK signaling cascade and enhances the secretion of reactive ROS and pro-inflammatory mediators, including IL-1β [96, 97]. Rac1 is implicated in several phases of *H. pylori* infection (Fig. 3); however, the precise mechanisms remain under research.

Lipopolysaccharide (LPS), vacuolating cytotoxin gene A (VacA), and cytotoxin-associated gene A (CagA) are the main factors that determine Helicobacter pylori’s virulence. Toll-like receptor 4 (TLR4) on gastric epithelial cells has been shown to recognize the endotoxin LPS, triggering a complex cascade of cellular signaling responses, including the MyD88-dependent and TRIF-dependent pathways [98, 99]. It functions as the main innate immune system detection and communication mechanism [100]. *H. pylori* LPS activates primary gastric mucosal cells, resulting in the induction of Dock180 phosphorylation and a notable elevation in Rac1-GTP levels [99]. LPS enhances NF-κB expression via the PI3K/Rac1/PAK1 signaling pathway, subsequently elevating IL-1β expression and facilitating pro-IL-1β maturation through the Rac1/PAK1/caspase-1 signaling pathway [101]. Additionally, it upregulates MMP-9 release through the Rac1/p38/cPLA2 signaling axis [102]. The LPS pathway also enhances the activation of Rac1 and NOX1 in AGS and NCI-N87 cell lines, increasing ROS concentrations [103]. Moreover, Rac1 promotes TNF-α converting enzyme in stomach mucosal cells [104]. These results indicate that Rac1 is pivotal in oxidative stress and inflammation related to gastric cancer.

CagA is tyrosine phosphorylated by Src family kinases inside cells, attaches itself to Crk adaptor proteins (Crk and Crk-L), triggers the Dock180/Rac1 signaling pathway, and facilitates cell cycle progression, motility, migration, and phagocytosis [28]. Additionally, Crk/Crk-L activates intracellular Abl (c-Abl), which in turn promotes migration and adhesion by stimulating actin rearrangements and cell scattering via the Cortical Y-470 Phosphorylation/GEF binding (Vav2 binding)/Rac1 GTP signaling pathway [53, 105].

The secreted multifunctional toxin VacA binds to the plasma membrane domain (lipid rafts) above the F-actin structure. Through lipid raft-dependent internalization, it reaches the late endocytic compartment and induces vacuolation [106]. VacA causes apoptosis by accumulating connexin 43 in autophagic vesicles through a glutathione (GSH)/Rac1/ERK-dependent mechanism [107].

Interestingly, Rac1 expression has also been reported to decrease in *H. pylori*-infected GES-1 gastric epithelial cells. In this context, altered actin polymerization was observed via the activation of the ILK/Rac1/PAK1 signaling pathway, facilitating pseudopod formation and cellular migration [108]. Moreover, Rac1 shows hypomethylation in *H. pylori*-infected gastric tumors, although its upstream regulators, ELMO1 and DOCK180, are consistently and substantially hypermethylated [109].

## Rac1 as a molecular therapeutic target in gastric cancer

### Molecular inhibitors of Rac1

The pervasive overexpression and hyperactivation of Rac1 in various treatment-resistant malignancies have made it an attractive target for therapy [110]. Initial studies identified NSC23766 as a first-generation Rac1 inhibitor that disrupts Rac1-GEF interactions (Trio/Tiam1 binding) while preserving Cdc42 and RhoA activity [111]. Subsequent efforts led to the development of EHop-016, which targets Rac GEF Vav. EHop-016 demonstrates PAK1 suppression and a lower half maximal inhibitory concentration (IC50) [112, 113]. Other Rac1-targeting compounds, including EHT 1864, Z62954982, and 1D-142, were all reported to inhibit Rac1 in tumor models [114–116]. More recently, R-ketorolac became the first FDA-approved Rac1/Cdc42 inhibitor and has been shown to significantly reduce cancer spread in ovarian cancer [117].

Additionally, small-molecule inhibitors targeting upstream and downstream effectors of Rac1 have been investigated. For instance, LY294002 (a PI3K inhibitor) and U0126 (a MEK/ERK inhibitor) reduce Rac1 activity by disrupting its upstream signaling, while SP600125 (a JNK inhibitor) diminishes Rac1 activation by influencing its downstream signaling [118–120].

To date, NSC23766 remains the only synthetic inhibitor specifically investigated in gastric cancer cells. Despite the availability of new molecular inhibitors, studies focusing on Rac1 inhibition in gastric cancer are still limited. However, several natural biological extracts have demonstrated Rac1-inhibitory activity in gastric cancer models (Table 3). For example, icariin, a purified extract from the Herba epimedium (a traditional Chinese medicine), adversely impacts the invasion and migration of gastric cancer cells through the Rac1-dependent vasodilator-stimulated phosphoprotein (VASP) pathway [121]. Nobiletin, a citrus flavonoid, inhibits Rac1 expression via the FAK/PI3K/Akt pathway, as well as Ras, c-Raf, Cdc42, and RhoA expression [122]. Diallyl disulfide (DADS), produced from garlic, has been demonstrated to inhibit the Rac1-Pak1/Rock1-LIMK1 pathway and obstruct TGF-β1/Rac1 signaling [83, 84]. Xu et al. discovered that the bioactive protein pPeOp (from Omphalia lapidescens) could reduce the expression and activation of Rac1 and Cdc42, along with their downstream targets (p-PI3K, p-AKT, AKT, p-PAK1, and RACGAP1), resulting in the disruption of cytoskeletal structure and the inhibition of gastric cancer cell proliferation, migration, and invasion [123].

**Table 3:** Rac1 inhibitors have been proven in gastric cancer

No.	Rac1 inhibitors	cell lines	Functions	Verification Type	Year	References
1	Icariin	BGC-823	Negatively affects tumor cell invasion and migration via the Rac1-dependent vasodilator-stimulated phosphoprotein (VASP) pathway.	in vitro	2010	[121]
2	Gallic acid (GA)	AGS	Inhibits NF-κB activity and downregulates the PI3K/AKT pathway, decreasing the expressions of Ras, cdc42, Rac1, RhoA, and RhoB.	in vitro	2010	[124]
3	Nobiletin	AGS	Inhibits the FAK/PI3K/Akt pathway while reducing the protein expression of Ras, c-Raf, Rac-1, Cdc42, and RhoA.	in vitro	2011	[122]
4	NSC23766	SGC-7901	Inhibits Rac1 binding and activates Rac-specific GEF Trio or Tiam1.	in vitro, in vivo, clinical samples	2015	[125]
5	Diallyl disulfide (DADS)	MGC803	Down-regulates the Rac1-Pak1/Rock1-LIMK1 pathway and reduces p-LIMK1 and p-cofilin1.	in vitro, in vivo, clinical sample	2016	[84]
		MGC803	Blocks TGF-β1/Rac1 signaling and down-regulates Rac1 and β-catenin.	in vitro, in vivo	2018	[83]
6	pPeOp	HGC-27	Up-regulates miR-30b-5p, suppresses RAB22A expression, and inhibits Rac1 and downstream molecules.	in vitro, clinical sample	2022	[123]

### miRNA-mediated Rac1 silencing mechanisms

MicroRNA (miRNA) dysregulation constitutes a hallmark of neoplastic transformation, mechanistically driving carcinogenesis through dual functionality as epigenetic modulators, exerting oncogenic or tumor-suppressive functions [126, 127]. Targeting specific miRNAs offers a promising cancer therapy approach by regulating genes linked to tumor growth, metastasis, TME, and immune response [128, 129].

Table 4 displays the miRNAs that inhibited Rac1 in gastric cancer. Reports indicate that miR-1296-5p, miR-630, miR-124-3p, miR-148b-3p, and miR-345 modulate Rac1 expression through several signaling axes. MiR-345 directly targets epidermal growth factor receptor pathway substrate 8 (EPS8), which leads to the inactivation of Rac1 and inhibits cell migration, EMT, and the cancer stem cell phenotype, thereby suppressing gastric cancer metastasis [130]. MiR-19a and miR-96 inhibit kinesin superfamily protein 26 A (KIF26A) expression via the FAK-PI3KR1-VAV3-Rac1-PAK3 axis, hence suppressing EMT and migration [131].

**Table 4:** miRNAs inhibit the Rac1 protein expression in gastric cancer

No.	Gene therapy	Cell lines	Functional pathway	Verification Type	Year	References
1	miR-1296-5p	SNU-216 and NUGC-4	Targets the ERBB2/Rac1 signaling pathway that inhibits migration and invasion of human gastric cancer cells.	in vitro, clinical samples	2017	[132]
2	miR-630	SGC-7901	Supposedly, via inhibits the Ras/PI3K/AKT pathway to regulate FoxM1 to suppress EMT.	in vitro	2017	[133]
3	miR-124-3p	SGC-7901 and MKN-28	Targets Rac1 and specificity protein 1 (SP1) to inhibit gastric cancer growth.	in vitro, in vivo, clinical samples	2018	[134]
4	miR-148b-3p	SGC-7901, AGS, HGC-27, MGC-803, and BGC-823	Inhibits the Dock6/Rac1/Cdc42 axis to decrease gastric cancer cell motility.	in vitro, in vivo, clinical samples	2018	[45]
5	miR-345	AGS and HGC-27	Inactivates Rac1 by targeting EPS8 to inhibit gastric cancer cell migration, EMT, and stem-like cell phenotypes.	in vitro, clinical samples	2020	[130]
6	RhoGDI2 miRNA	MGC-823 and SGC-7901	Downregulates gastric cancer cell migration and invasion by attenuating the EMT cascade via the Rac1/Pak1/LIMK1 pathway.	in vitro, in vivo, clinical samples	2020	[135]
7	miR-19a and miR-96	BGC823 and SGC7901	Inhibit the KIF26A expression and inactivate the FAK/PI3KR1/VAV3/Rac1/PAK3 axis to suppress gastric cancer cell migration and invasion.	in vitro, in vivo, clinical samples	2021	[131]
8	miR-1915-3p	SGC-7901, MKN-45, MGC-803, BGC-823, and HGC-27	Inhibits Rac1 expression to inhibit gastric cancer cell growth.	in vitro, in vivo, clinical samples	2022	[136]
9	miR-30b-5p	HGC-27	Diminishes the expression and activation of Rac1/Cdc42 by targeting RAB22A, hence altering the architecture of microfilaments and microtubules and reducing lamellipodia formation.	in vitro, clinical samples	2022	[123]

### Immunotherapy of Rac1

Immunotherapy is crucial for the conservative treatment of advanced gastric cancer. Rac1 expression in tumor cells maintains an immunosuppressive state that lowers patient response rates to immune checkpoint inhibitors by positively correlating with Th2 cell and macrophage invasion in the tumor microenvironment [137, 138]. Nonetheless, its potential as an immunotherapeutic target in gastric cancer remains largely unexamined, signifying a promising avenue for further investigation. This section provides a brief overview of the current state of Rac1-related research in immunotherapy.

The expression of Rac1 in the immune system often influences the growth of immune cells and the construction of the immune microenvironment. Rac1 therefore has a great deal of promise for improving immune response and tackling medical issues such as tumor treatment resistance. The expression of Rac has been shown to be involved in the development of common lymphoid progenitors (CLPs), Akt activation, IL-2 production, and T cell maturation and proliferation in thymocytes. Its absence significantly reduces T cell adhesion, migration, and survival [139]. In Kit 225 T cells, glycogen phosphorylase muscle isoform (PYGM) can control Rac1 activity, allowing IL-2-stimulated T cell proliferation [140]. Similarly, Rac1 loss has been associated with similar deficiencies in B cell receptor proliferation, survival, adhesion, and migration [141].

In melanoma, increased Rac1 activation promotes cytotoxic T-cell (CTL) homing to lymph nodes and tumors, alongside increased proliferation [142], whereas Rac1^P29S^ mutations regulate PD-L1 expression [143]. In ulcerative colitis-associated cancer, Rac1 influences neutrophil chemotaxis and apoptosis [144]. Rac1 also complexly impacts ovarian tumor microenvironment signaling [145]. Moreover, Rac1 GEF DOCK4 exhibited a substantial correlation with the expression of prognostic immunological biomarkers in stomach adenocarcinoma (STAD), potentially implicating it in immune infiltration and immune evasion [55].

## Conclusions and perspectives

Rac1, a key member of the Rho GTPases family, regulates cytoskeletal dynamics, cellular adhesion, and motility [12, 32, 146]. Its activity is strongly associated with gastric cancer progression and contributes to critical processes such as cell proliferation, EMT, migration, and invasion. Emerging evidence also implicates Rac1 in modulating the tumor immune microenvironment, highlighting its relevance beyond traditional oncogenic roles. Importantly, Rac1 activity may vary depending on tumor cell subtype, microenvironmental conditions, and cross-talk with other signaling pathways. A deeper understanding of the multifaceted roles of Rac1 will be essential for developing effective, biomarker-driven therapeutic strategies for gastric cancer.

The intricate regulation of Rac1 activity entails complex interactions across multiple signaling networks and varies among distinct molecular subtypes of gastric cancer, complicating its therapeutic targeting. The Lauren classification categorizes gastric cancer into intestinal-type gastric cancer (IGC) and diffuse-type gastric cancer (DGC) [147]. Most DGCs belong to the genomic stability type and exhibit a high rate of peritoneal metastasis [148]. Zhang et al. established cell lines exhibiting biological behaviors similar to those of IGCs and DGCs and found that Rac1 inhibitors significantly suppressed the motility of DGC-like cells but failed to inhibit another type [15].

Off-target effects and pathway redundancy can further obfuscate the clinical efficacy of Rac1 inhibitors. Therefore, deeper mechanistic studies are essential to clarify Rac1’s role in gastric cancer progression and to map its interactions with key oncogenetic pathways. Notably, recent findings on MAPK1 have broadened this perspective. Beyond its canonical kinase function, MAPK1 also acts as a bidirectional transcription factor that binds directly to gene promoters, coordinately upregulating or downregulating genes involved in cell motility and invasion [149]. This dual role underscores the expanding landscape of transcriptional regulators in gastric cancer metastasis. In parallel, integrative genomics and transcriptomic analyses of Rac1-related gene expression profiles in gastric cancer tumor tissues may improve patient stratification and enhance the development of more precise, targeted therapeutic strategies.

The application of Rac1 inhibitors in tumor therapy remains a subject of significant interest. Hemsing et al. found that combining EHop-016 with doxorubicin (DNR) significantly reduced tumor burden in zebrafish larvae [150]. All currently available Rac1 inhibitors present notable limitations, including low specificity and inappropriate IC50, which may lead to significant side effects in clinical applications. Research has also indicated that the Rac1 inhibitor 1A-116 can cross the blood-brain barrier, exhibits favorable toxicological properties, and demonstrates a dose-dependent antitumor effect in a human glioblastoma nude mouse model [151]. Research on Rac1-targeted therapeutic strategies for gastric cancer remains limited, largely due to the complex signaling cross-talk within the Rho GTPase network and unresolved pharmacodynamic challenges related to selectively modulating Rac1-driven oncogenic pathways.

Emerging evidence shows that Rac1-targeting miRNAs can suppress oncogenes in gastric cancer cells, thereby inhibiting proliferation, EMT, and invasion. Future studies should focus on elucidating the regulatory roles of these miRNAs in epigenetic modification, gene silencing, and protein signaling. These insights may contribute to the molecular subtyping of gastric cancers and facilitate the development of miRNA-based targeted therapies aligned with specific oncogenic profiles. Combination therapy strategies with Rac1 inhibitors have been shown to significantly reduce cancer cells’ proliferation and migration capacity and restore tumor chemosensitivity [152–154]. In other tumors, Rac1 inhibition has also shown positive effects in enhancing the ability of immune cells in immune combination therapy [155–157]. However, Rac1 inhibitors have not yet been studied in combination with other signal molecule inhibitors and immuno-related therapy in gastric cancer, both of which are expected to become hotspots for future research. In the gastric cancer spontaneous lung metastasis model, the combination of 5-FU and a traditional Chinese medicine regimen implicates the potential of Rac1 inhibition in preventing gastric cancer distinct metastasis [158].

In conclusion, mounting experimental data supports Rac1 as a key regulator of the pathophysiological processes in gastric cancer development and metastasis, presenting significant promise as a novel therapeutic target. Through deeply studying its regulatory mechanisms, investigating its clinical applications, and integrating laboratory results with clinical research, we anticipate the development of more efficacious therapy regimens that will enhance the prognosis for gastric cancer patients.

## Abbreviations

ARHGAP15: a Rho GTPase activating protein 15
CRIB: Cdc42-Rac-interacting-binding
CD: Cluster of differentiation
CLPs: Common lymphoid progenitors
CagA: Cytotoxin-associated gene A
NEDD9: Neural precursor cell expressed developmentally downregulated protein 9
DADS: Diallyl disulfide
DGC: Diffuse-type gastric cancer
EPS8: Epidermal growth factor receptor pathway substrate 8
EMT: Epithelial-to-mesenchymal transition
GC: Gastric cancer
GSK3β: Glycogen synthase kinase 3β
GAPs: GTPase-activating proteins
GDIs: Guanine nucleotide dissociation inhibitors
GEFs: Guanine nucleotide exchange factors
H. pylori: Helicobacter pylori
HIF: Hypoxia-inducible factor
IGC: Intestinal-type gastric cancer
IQGAP1: IQ motif-containing GTPase-activating protein 1
IRSp53: Insulin receptor tyrosine kinase substrate p53
KIF26A: Kinesin superfamily protein 26 A
LIMK: LIM kinase
MMP: Matrix metalloproteinases
miRNA: MicroRNA
MICAL1: Molecule Interacting with CasL1
NOX: NADPH oxidase
PAK: p21-activated kinase
PLC: Phospho-inositide-specific phospholipase C
PRK1: Protein kinase C-related kinase 1
Rac1: Ras-related C3 botulinum toxin substrate 1
ROS: Reactive oxygen species
RhoGDI2: Rho GDP dissociation inhibitor 2
Rho GTPases: Rho guanosine triphosphatases
sFRP1: Secretory frizzled-related protein 1
STAT3: Signal transducers and activators of transcription 3
Smad3: SMAD family member 3
STAD: Stomach adenocarcinoma
TIMP: tissue inhibitor of metalloproteinases
VacA: Vacuolar cytotoxin
VEGF: Vascular endothelial growth factor
VASP: Vasodilator-stimulated phosphoprotein
WASP: Wiskott-Aldrich Syndrome Protein
WAVE: WASP-family verprolin-homologous
WCA: WH2-central-acidic
WRC: WAVE Regulatory Complex
ZEB1: Zinc finger E-box-binding homeobox 1
